# Impulsivity and non-suicidal self-injury in adolescents: a systematic review and meta-analysis of longitudinal studies

**DOI:** 10.3389/fpsyt.2025.1586922

**Published:** 2025-05-21

**Authors:** Xubin He, Ping Huang, XingHong Xu, Qinyao Yu, HongYan Huang, Ping Yang, Bo Yang

**Affiliations:** ^1^ Chongqing Mental Health Center, Chongqing, China; ^2^ Chongqing Medical School, Chongqing, China

**Keywords:** impulsivity, non-suicidal self-injury, adolescents, longitudinal studies, meta-analysis

## Abstract

**Background:**

Non-suicidal self-injury (NSSI) is a prevalent and concerning behavior among adolescents, with impulsivity commonly considered an important risk factor. However, the strength of this relationship remains unclear. This systematic review and meta-analysis aim to assess the relationship between impulsivity and NSSI in adolescents, focusing on longitudinal studies.

**Methods:**

A comprehensive literature search was conducted across seven databases, with the search extending to February 1, 2025, to identify longitudinal studies on impulsivity and NSSI in adolescents. The effect sizes (odds ratio, OR) for each study were calculated, and a meta-analysis was performed to synthesize the results. Subgroup analyses were also conducted to examine whether the association between impulsivity and NSSI was influenced by factors such as region, age, impulsivity measurement tools, NSSI measurement tools, follow-up period, and study quality. A fixed-effect model was used to assess differences in effects across subgroups. All statistical analyses were performed using STATA 16.0 software.

**Results:**

Nine longitudinal studies involving 33,973 participants were included in the meta-analysis. A statistically significant positive correlation was found between impulsivity and NSSI in adolescents. The OR for NSSI was 1.09 (95% *CI*: 1.04, 1.16). Subgroup analyses revealed that Asian adolescents (OR = 1.18, 95% *CI*: 1.03, 1.36) and middle school students (OR = 1.11, 95% *CI*: 1.02, 1.20) were at a higher risk. These findings underscore the importance of targeting interventions towards these at-risk groups.

**Conclusion:**

This meta-analysis suggests that impulsivity is a significant predictor of adolescent NSSI. The findings highlight the importance of early identification of impulsive behaviors in high-risk adolescent groups, particularly in populations such as adolescents from Asia and middle school students.

**Systematic review registration:**

https://www.crd.york.ac.uk/prospero/display_record.php?ID=CRD42025641716, identifier CRD42025641716.

## 1 Introduction

Non-suicidal self-injury (NSSI) refers to the deliberate and repetitive self-harm without clear suicidal intent, commonly manifested in behaviors such as cutting, burning the skin, hitting oneself, or banging against walls. These actions are generally non-lethal and not accepted by society (1). Adolescents represent a high-risk group for NSSI, with its prevalence showing an increasing trend over time (2). Research has indicated that the incidence of NSSI peaks at ages 14 to 15 and subsequently declines around age 18 (3). A study of 64,671 U.S. community adolescents found the prevalence of NSSI to be 17.6% (4). A joint survey conducted across 11 European countries (Germany, Sweden, Italy, France, Spain, etc.) involving 12,068 school-aged adolescents revealed a prevalence range between 17.1% and 38.6% (5). However, adolescents in Asia have a higher prevalence of NSSI compared to those in other continents (14.7% vs. 19.5%) (6). Currently, NSSI has become increasingly prevalent among adolescents, posing a significant public health issue that affects their mental health and may result in physical harm (7). NSSI not only increases the risk of depression, anxiety, substance abuse, and recurrent self-harming behaviors, but it also significantly raises the likelihood of suicidal ideation and behaviors (8, 9). Individuals who engage in NSSI have a 4.27-fold increased risk of suicidal ideation and a 1.51-fold increased likelihood of death by suicide, according to a meta-analysis of longitudinal studies (10). The interpersonal theory of suicide suggests that NSSI serves as a short-term response to suicidal thoughts. Through repeated self-injury, individuals may gradually build a higher tolerance to self-harm, potentially increasing their suicide risk (11). Therefore, addressing adolescent NSSI and evaluating its influencing factors is of crucial importance for the prevention of NSSI and the promotion of adolescent mental and physical health.

Impulsivity is characterized by the tendency to react quickly and without careful thought to both internal and external stimuli, often ignoring the potential adverse effects these reactions might have on oneself or others (12). Impulsivity is a multifaceted construct that can be assessed using various methods. Previous studies have identified three categories of impulsivity: impulsive decision-making, impulsive behavior, and impulsive personality traits (13). Impulsive personality traits are commonly evaluated through self-report instruments, including the barratt impulsiveness scale and the UPPS-P impulsive behavior scale (14, 15). Impulsive behavior and impulsive decision-making are considered state impulsivity, influenced by environmental factors, and are assessed using laboratory-based behavioral tasks (16, 17). Impulsivity is widespread in the population and contributes to core personality traits such as novelty-seeking, sensation-seeking, and extraversion (18). However, numerous studies have shown that impulsivity is associated with problematic behaviors, including attention deficit hyperactivity disorder (19), substance abuse (20), and NSSI (21). For a long time, NSSI has been commonly regarded as a symptom of borderline personality disorder, with impulsivity being a core feature (22). Hawton et al. (23) suggest that the onset of NSSI results from a combination of various interrelated elements, including biological, psychological, genetic, cultural, and social factors. High impulsivity and other personality traits have been shown to be closely related to NSSI (24). The urgency theory suggests that individuals are more likely to engage in impulsive behaviors, such as NSSI, when negative emotions are intensified (25). Previous research has confirmed that impulsivity and negative emotions mutually influence adolescent NSSI behaviors (26, 27). A longitudinal study found that increased impulsivity is a potential risk factor for NSSI and can independently predict the onset of new NSSI cases (28). However, there are inconsistent findings regarding the relationship between impulsivity and NSSI, with some studies reporting a significant association (29), while others have failed to find such a relationship (30). Therefore, studying impulsivity in adolescents is an important avenue for understanding the causes of self-injury and for developing targeted clinical interventions.

To date, four systematic reviews have extensively explored the relationship between impulsivity and NSSI, one of which is a narrative review (31). While these reviews support the existence of some form of relationship, the specific details and strength of this relationship remain unclear, primarily due to methodological limitations in the studies reviewed. Another systematic review found a significant positive correlation between impulsivity and NSSI, but it did not delve into the specific situation of adolescents (32). Furthermore, two recent systematic reviews (33, 34) have also emphasized the association between impulsivity and NSSI, with this relationship being particularly pronounced. However, these reviews only analyzed the neurobehavioral or neurocognitive features of impulsivity in relation to NSSI and did not address studies that measured impulsivity using scales. At the same time, much of the previous research has primarily included cross-sectional observational designs, which can only reveal correlations between impulsivity and NSSI, without providing clear evidence for causal relationships. Therefore, this study aims to integrate the findings of earlier research and employs a longitudinal research design to further explore the causal relationship between impulsivity and NSSI among adolescents. Additionally, sociodemographic characteristics (such as age and region), follow-up time, variations in assessment instruments, and study quality are the primary focus of this investigation. This strategy aims to bridge the gaps in current research and lay the theoretical groundwork for future intervention techniques that are more specifically tailored to the needs of the population. In conclusion, this study will address the following research questions: (1) How strong is the correlation between teenage impulsivity and NSSI? (2) How is the correlation between impulsivity and NSSI moderated by variations in study methodologies’ handling of variables?

## 2 Methods

### 2.1 Materials and methods

All of the procedures for conducting the review, from determining who was eligible to collecting and analyzing data, were laid out in advance in the review protocol and recorded in PROSPERO (CRD42025641716). All procedures for conducting systematic reviews were adhered to in this study (35).

### 2.2 Search strategy

We implemented a comprehensive search strategy to identify studies relevant to this meta-analysis. The strategy focused on identifying longitudinal studies exploring the relationship between impulsivity and NSSI. The search was conducted across seven electronic databases, including Embase, PubMed, Web of Science, Wanfang Data, CNKI, VIP Database, and SinoMed, with no restriction on publication date, aiming to maximize the identification of potentially relevant literature. The search was limited to studies published until February 1, 2025. The search employed predefined keyword combinations related to impulsivity, NSSI, and longitudinal study design. Boolean operators (AND, OR) were used to combine these keywords and broaden the search while maintaining relevance. Specific search terms included: Impulsivity: “impulsivity”, “impulsive behavior”, “impulsive traits”, “UPPS”, “Sensation Seeking”, “Negative Urgency”, “Premeditation”, “Perseverance”, “Positive Urgency”, “Barratt Impulsivity Scale”, “Eysenck Personality Questionnaire”, and others. NSSI: “non-suicidal self-injury”, “self-harm”, “self-injurious behavior”, “self-mutilation”, “deliberate self-harm”, “self-inflicted injury”, “nonsuicidal self-injury”, “self-cutting”, and related terms. Longitudinal studies: “longitudinal study”, “prospective study”, “follow-up study”, “trajectories”, “course”, “time point”. Additionally, we also looked for additional research by hand in the reference lists of the included studies and the reviews that were relevant to the topic. PH read only the abstracts and titles of the publications, while XHX and HYH evaluated the entire texts using established criteria. The **Supplementary Materials** provide more information about the search approach.

### 2.3 Study selection criteria

Inclusion criteria: (1) Study design: The study design must be cohort-based, aiming to explore the causal relationship between the exposure factor (impulsivity) and the outcome (NSSI). (2) Population characteristics: Participants should be community adolescents aged 10 to 24 years (36), including those with data collected during childhood, with the follow-up period spanning the entire 10 to 24 years age range. (3) Outcome: The study must report NSSI-related outcome variables and provide relevant statistical data (e.g., odds ratio [OR]). When data from different studies on the same cohort are reported at different time points, the study with the longest follow-up period will be selected. (4) Follow-up duration: The study must have a follow-up period of at least 3 months, providing data on the effect of impulsivity on NSSI. (5) Control variables: The study must account for and report the impact of controlling for confounding variables (e.g., gender, age, etc.) on the results of the analysis. (6) Study publication: The study must be published in either English or Chinese. (7) Publication period: Studies published up to February 1, 2025, were included to maximize the identification of relevant literature. Only studies published within this period were considered for inclusion in this meta-analysis.

Exclusion criteria: (1) Study design: Only cohort studies will be considered. No cross-sectional or case-control studies will be considered. (2) Population characteristics: Exclude studies where the participants’ age falls outside the target age range for the meta-analysis (i.e., not between 10 and 24 years) or where insufficient demographic information is provided. (3) Outcome: Exclude studies that do not measure NSSI or fail to report sufficient statistical data (e.g., OR) to calculate effect sizes. (4) Follow-up duration: Exclude studies that do not offer valid follow-up data or have a follow-up period of less than 3 months. (5) Exposure factor: Consideration of exposure: research in which the association between impulsivity and NSSI is not well established should be excluded. (6) Study quality: Researchers should not include studies that are highly biased or that do not account for important confounding factors when evaluating their quality. (7) Study type: Review articles, meta-analyses, abstracts from conferences, letters, comments, case reports, and any other sort of non-original cohort study are not eligible.

### 2.4 Data extraction

In order to guarantee the included studies’ trustworthiness and consistency, the data extraction process was carried out meticulously according to the stated protocol. Data extraction was independently conducted by two reviewers (XBH, QYY) following a standardized protocol, and it was arranged using Excel 2021. Included in this data set were the following pieces of information: study details (authors, year of publication, and region), sample demographics (age range, proportion of females, baseline and final sample sizes, and duration of follow-up), and instruments used to measure impulsivity and NSSI. Also reported were important findings, such as the correlation between impulsivity and NSSI outcome markers, along with OR and 95% *CI*. If essential data were missing, study authors were contacted via email for clarification. Where no response was received, sensitivity analysis was conducted to assess the impact of missing data. In addition, the total effect size for impulsivity was calculated by aggregating data for studies that used various subscales to measure impulsivity. To do this, we took the effect sizes of each subscale and averaged them, using the sample size as the weight. The meta-analysis then used the aggregated effect size to determine the total influence of impulsivity on NSSI. Where possible, all variables that could have influenced the results were considered during data collection. Use of NoteExpress was made for the purpose of preliminary literature screening and removal of duplicate data. A third reviewer thoroughly examined all of the extracted data to guarantee its accuracy (PY). When reviewers disagreed, they met to discuss the matter and eventually come to a mutual agreement. In cases where a unanimous judgment was not possible, a senior researcher (BY) was contacted for the last say.

### 2.5 Quality assessment

To evaluate the included studies’ quality, we utilized the Newcastle-Ottawa Scale (NOS) for cohort studies. Eight items make up this scale, with a total score ranging from 0 to 9 stars. Four items evaluate the selection of participants, one evaluates the comparability of participant groups, and three examine the outcomes (37). This study used the following quality ranking system: Studies were classified as low-quality if they scored less than 4 stars, as high-quality studies were assigned 7–9 stars, moderate-quality research were given 4–6 stars, and so on. In order to guarantee scientific rigor, we exclusively considered papers that received four stars or higher. Each of the listed studies was evaluated independently by two reviewers (XBH and PY) for quality. To ensure the quality assessment procedure was accurate and consistent, any disagreements between the reviewers were either discussed or, in the event that they could not be addressed, a third reviewer (BY) was brought in.

### 2.6 Statistical analysis

We computed the pooled OR and its 95% Confidence Interval (*CI*) using the fully adjusted ORs from the included studies to evaluate the association between impulsivity and NSSI in adolescents. Heterogeneity was assessed using Cochran’s Q test and the *I²* statistic. Heterogeneity was considered significant if *I²* > 50% or the *P*-value from Cochran’s Q test was less than 0.05. The fixed-effect model disregards study-to-study variation, assuming that all studies share the same true effect size and attributing any observed variance solely to sampling error. The random-effects model, instead of assuming a single true effect size, accounts for both sampling error and between-study variation to explain heterogeneity, making it preferable to the fixed-effect model. The pooled effect size is a weighted average of the true effect sizes from all studies, which justifies the use of the random-effects model (38). We conducted sensitivity analyses by reanalyzing the data with different statistical models and excluding studies with a high risk of bias to ensure the robustness of our findings (39). To further evaluate the potential for publication bias, we performed Egger’s regression test following an initial examination with funnel plot analysis (40, 41). The trim-and-fill method was applied to correct for significant publication bias, if detected (42). We sub-grouped factors such as region, age, measurement tools for impulsivity and NSSI, follow-up period, and study quality to assess their impact on the association between impulsivity and NSSI. The effect differences between the subgroups were evaluated using fixed-effect models. We used STATA 16.0 (StataCorp, College Station, TX, USA) for all of our statistical analyses. The level of statistical significance was set at *P* < 0.05.

## 3 Results

### 3.1 Attributes and quality assessment of the included research

**Figure 1:**
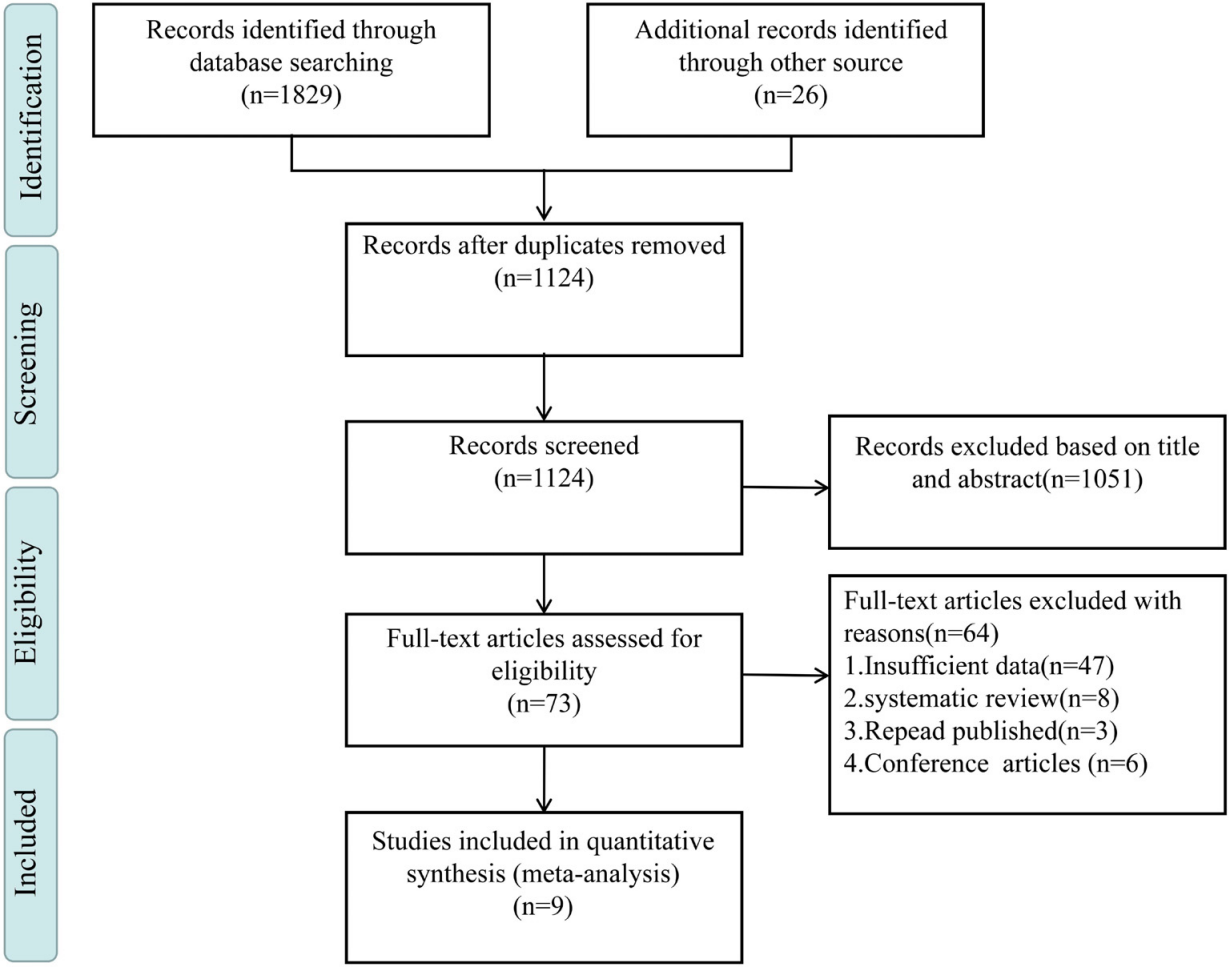
Literature selection process.

We found a total of 1,829 items. Only 73 full-text items remained for eligibility assessment after abstracts and duplicates were removed. 64 records were excluded for the following reasons: no data extraction (n = 45), systematic reviews (n = 8), duplicate publications (n = 5), or conference papers (n = 6). Ultimately, nine articles that met the specified inclusion criteria were selected (29, 43–50). The record search procedure is shown in **Figure 1** via the PRISMA flow diagram. The studies, with an average age of 14.6–19.1 years and a sample size ranging from 559 to 14,062 participants, were published between 2009 and 2023. Asian countries accounted for approximately one-third of the studies (3/9). The follow-up period ranged from 3 months to 192 months. Regarding NSSI measurement tools, 5 studies (29, 44, 47, 49, 50) used well-established tools with validated psychometric properties in adolescent samples. These tools listed a range of behaviors that participants could use to identify whether they had engaged in self-injurious behaviors. The remaining 4 studies (43, 45, 46, 48) used measurement tools that had not been validated for reliability or validity. Of these, 2 studies (43, 46) assessed NSSI through self-report, while the other 2 (45, 48) used tools based on the self-harm issues included in the European Child and Adolescent Self-Harm (CASE) studies. In terms of impulsivity measurement tools, except for 1 study (45), which used the Stop-Signal Task (mean correct trial count), the other 8 studies (29, 43, 44, 46–50) used validated tools for assessing impulsivity. All studies included in the meta-analysis adjusted for potential confounders. As shown in the **Supplementary Materials**, a detailed quality assessment was conducted. Based on the NOS quality assessment criteria, 5 studies were rated as high quality, demonstrating rigorous and consistent research design, sample selection, exposure assessment, and outcome measurement. The remaining 4 studies were rated as moderate quality. Although these studies largely met the standards in terms of study design and execution, they exhibited limitations in certain areas, such as sample selection or the accuracy of exposure assessment. **Table 1** summarizes the main characteristics of these studies.

**Table 1:** Characteristics of the included article.

References	Region	Baseline /final sample size	Age range /Population /Female (%)	Follow-up period (Months)	OR (95%*CI*)	Type of assessment (NSSI)	Type ofassessment (Impulsivity)	Variables controlled	Study quality scores
O’Connor et al., 2009 (43)	United Kingdom	737/500	15.20 ± 0.70/M	6	1.16 (0.96, 1.40)	CASE	PIS	Sexual orientation worries, History of sexual abuse, Self-harm by family, Anxiety, Self-esteem.	6
You and Leung. 2012 (44)	China	6212/4782	14.56 ± 1.81/M /68.5	24	1.42 (1.31, 1.53)	DIB-R	DIB-R	Age, Depressive symptoms, Family invalidation, Behavioral impulsivity.	7
Mars et al., 2014 (45)	United Kingdom	14,062/4799	16.00/M/58.9	192	1.00 (0.96, 1.05)	CASE	SST	Gender, Socioeconomic position, IQ, Childhood sexual abuse, Parental cruelty to children, Being bullied, Impulsivity, Sensation-seeking, Body dissatisfaction, Depression, Anxiety disorder, Substance use, Self-harm in friends and family.	7
Riley et al., 2015 (29)	United States	1158/869	18.04/C/100	9	1.05 (0.86, 1.28)	DSHI	UPPS-P	Negative urgency, Lack of perseverance, Positive urgency, Lack of planning, Sensation seeking, Prior NSSI behavior.	6
Huang et al., 2017 (46)	China	5879/4331	16.02 ± 0.52/M /56.73	12	1.03 (1.00, 1.05)	Self-report	BIS-11	Sociodemographic data, Social support, Family discord, Impulsivity, Alcohol use, Tobacco use, Suicidality, Depressive symptoms, Self-esteem.	7
Hamza et al., 2019 (47)	Canada	1132/782	19.11 ± 1.05/C /71	24	1.03 (1.00, 1.05)	ISAS	BIS-11	Age, Sex, Parental education, born in Canada, Depressive symptoms, Anxiety, Emotion dysregulation.	7
Lockwood et al., 2020 (48)	United Kingdom	646/594	M/47	3	1.08 (0.99, 1.18)	CASE	SUPPS-P	Sex, Year group, Anxious, Depressive symptomatology, Emotion dysregulation, Positive and Negative Affect, Negative Urgency, Lack of Perseverance, Lack of Premeditation, Sensation-Seeking, Positive Urgency.	6
Dale et al., 2023 (49)	United States	559/458	15.25 ± 0.41/M /46	144	0.61 (0.37, 1.02)	K-SADS-PL	CBQ	Sadness, Anger, Impulsivity, Sex, Temperament.	9
Wang et al., 2023 (50)	China	3588/2527	16.13 ± 0.79/M /51.8	12	1.15 (1.09, 1.20)	FASM	DMSC	Age, Grade, Gender, Ethnicity, Single-child, Left-behind child, Home locality, Education level of father, Education level of mother.	6

M, Middle school; C, College; NSSI, Non-Suicidal Self-Injury; PIS, Plutchik Impulsivity Scale; DIB-R, Revised Diagnostic Interview for Borderlines; SST, Stop-Signal Task; CASE, Child and Adolescent Self-harm in Europe; UPPS-P, UPPS-P Impulsivity Scale; DSHI, Deliberate Self-Harm Inventory; BIS-11, Barratt Impulsiveness Scale Version 11; ISAS, Inventory of Statements About Self-Injury; SUPPS-P, Short UPPS-P Impulsivity Scale; CBQ, Child Behavior Questionnaire–Impulsivity Subscale; K-SADS-PL, Schedule for Affective Disorders and Schizophrenia for School-Age Children–Present and Lifetime Version; DMSC, Dual-Modes of Self-Control Scale–Impulse System Subscale; FASM, Functional Assessment of Self-Mutilation; OR, Odds Ratio; 95% *CI*, 95% Confidence Interval.

### 3.2 Homogeneity test and meta-analysis

#### 3.2.1 Evaluation of the Impulsivity-NSSI relationship through homogeneity testing and meta-analysis

**Figure 2:**
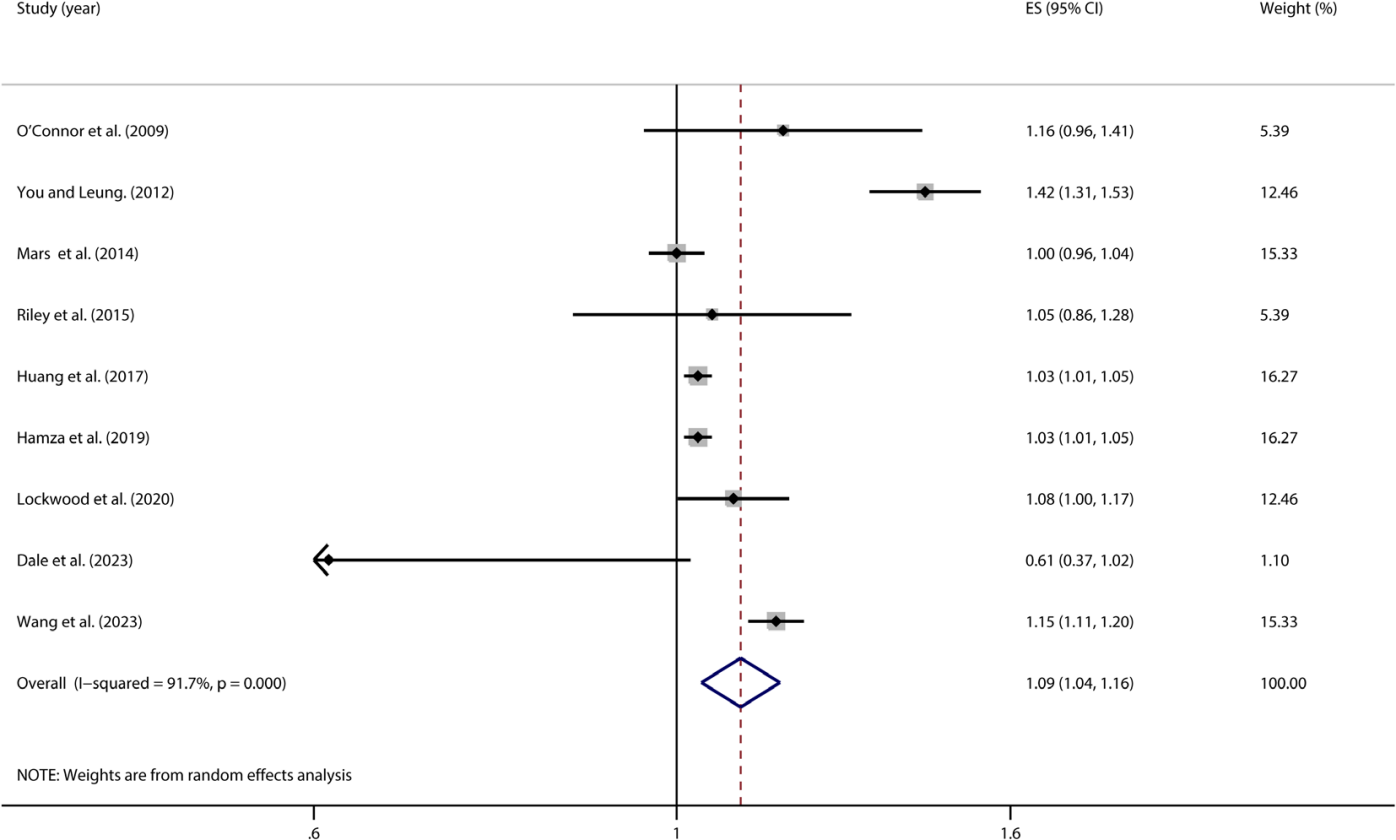
Forest plot of the relationship between impulsivity and NSSI.

The homogeneity test of the 9 studies included in this research showed high heterogeneity (*I²* = 91.7%, *P* < 0.001). Due to the considerable variability between studies, a random-effects model was applied to estimate the overall effect. The model analysis results indicated that the pooled effect size was OR = 1.09 (95% *CI*: 1.04, 1.16), and the association was statistically significant (*Z* = 3.19, *P* = 0.001). This suggests that adolescents with higher impulsivity have a 9% higher risk of engaging in NSSI compared to those with lower impulsivity (**Figure 2**).

#### 3.2.2 Comparison of impulsivity and NSSI through subgroup analysis

The subgroup analysis in this study explored the influence of region, age, impulsivity measurement tools, NSSI measurement tools, follow-up period, and study quality on the relationship between impulsivity and NSSI, with detailed results shown in **Table 2**.

**Table 2:** Subgroup analysis showing OR of NSSI for Impulsivity.

Variables	95% *CI* for OR		Heterogeneity test		Heterogeneity between the groups (*P*-value)
	*OR*	*95% CI*	*I^2^* (%)	*P*-value	
Region					0.002
Asians(n=3)	1.18	1.03, 1.36	97.40	< 0.001	
Non-Asians(n=6)	1.03	0.99, 1.07	45.60	0.102	
Age					0.037
Middle school student(n=7)	1.11	1.02, 1.20	93.50	< 0.001	
College student(n=2)	1.03	1.01, 1.05	00.00	0.842	
Impulsivity measurement tools					0.014
Unvalidated measures tool(n=1)	1.00	0.96, 1.04	—	—	
Validated measures tool(n=8)	1.11	1.04, 1.18	92.30	< 0.001	
NSSI measurement tools					0.002
Unvalidated measures tool(n=4)	1.03	1.00, 1.06	42.00	0.159	
Validated measures tool(n=5)	1.12	0.99, 1.27	95.10	< 0.001	
Follow-up period					0.219
≤1years(n=5)	1.09	1.02, 1.17	84.40	< 0.001	
>1years(n=4)	1.09	0.96, 1.24	95.70	< 0.001	
Quality studies					< 0.001
High-quality studies(n=5)	1.08	1.01, 1.16	94.30	< 0.001	
Fair-quality studies(n=4)	1.13	1.10, 1.17	00.00	0.485	

NSSI, Non-Suicidal Self-Injury.

**Figure 3:**
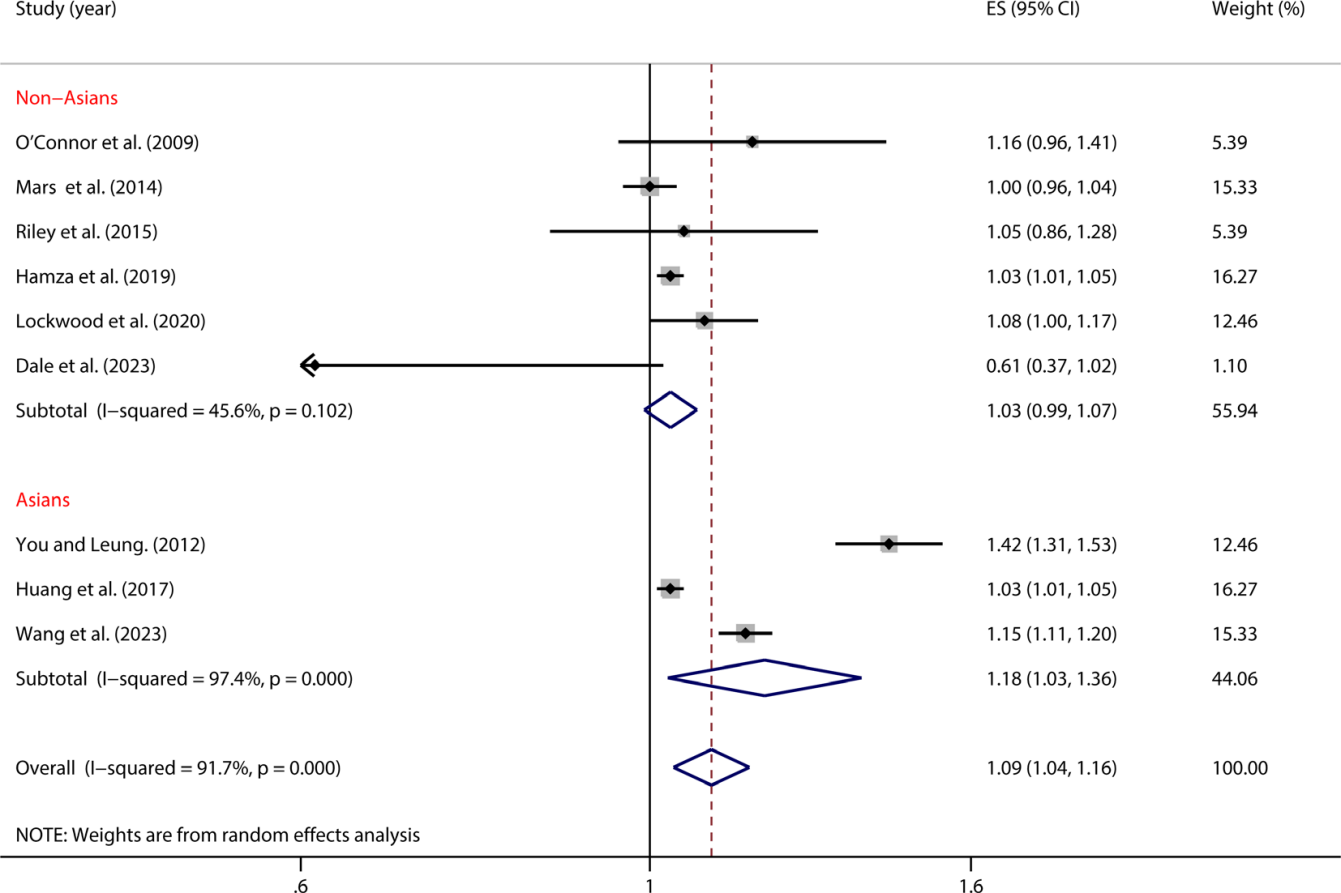
Moderation effect of regional differences on impulsivity and NSSI.

As shown in **Figure 3**, in the Asian sample, impulsivity was significantly associated with NSSI (OR = 1.18, 95% *CI*: 1.03, 1.36). However, in the non-Asian sample, there was no significant association between impulsivity and NSSI (OR = 1.03, 95% *CI*: 0.99, 1.07). The between-group difference was significant (*P* = 0.002), indicating that region might be an important moderating factor influencing the relationship between impulsivity and NSSI (**Table 2**).

**Figure 4:**
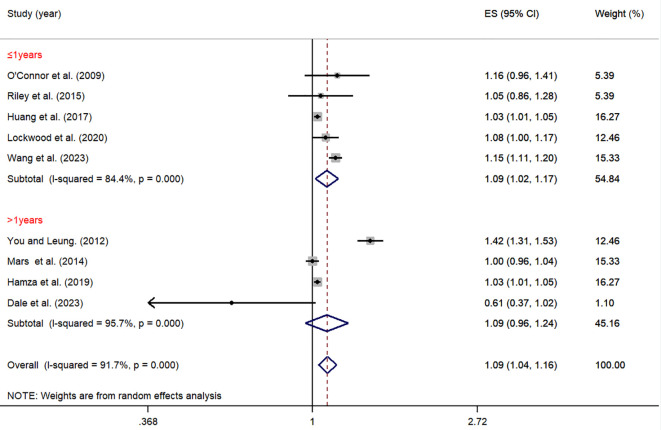
Moderation effect of age on impulsivity and NSSI.

As shown in **Figure 4**, In the middle school student group, impulsivity was significantly associated with NSSI (OR = 1.11, 95% *CI*: 1.02, 1.20), suggesting that impulsivity may be an important predictor of NSSI in this age group. However, in the university student group, the association between impulsivity and NSSI was weaker (OR = 1.03, 95% *CI*: 1.01, 1.05). The between-group difference was significant (*P* = 0.037), indicating that age may play an important moderating role in the relationship between impulsivity and NSSI (**Table 2**).

**Figure 5:**
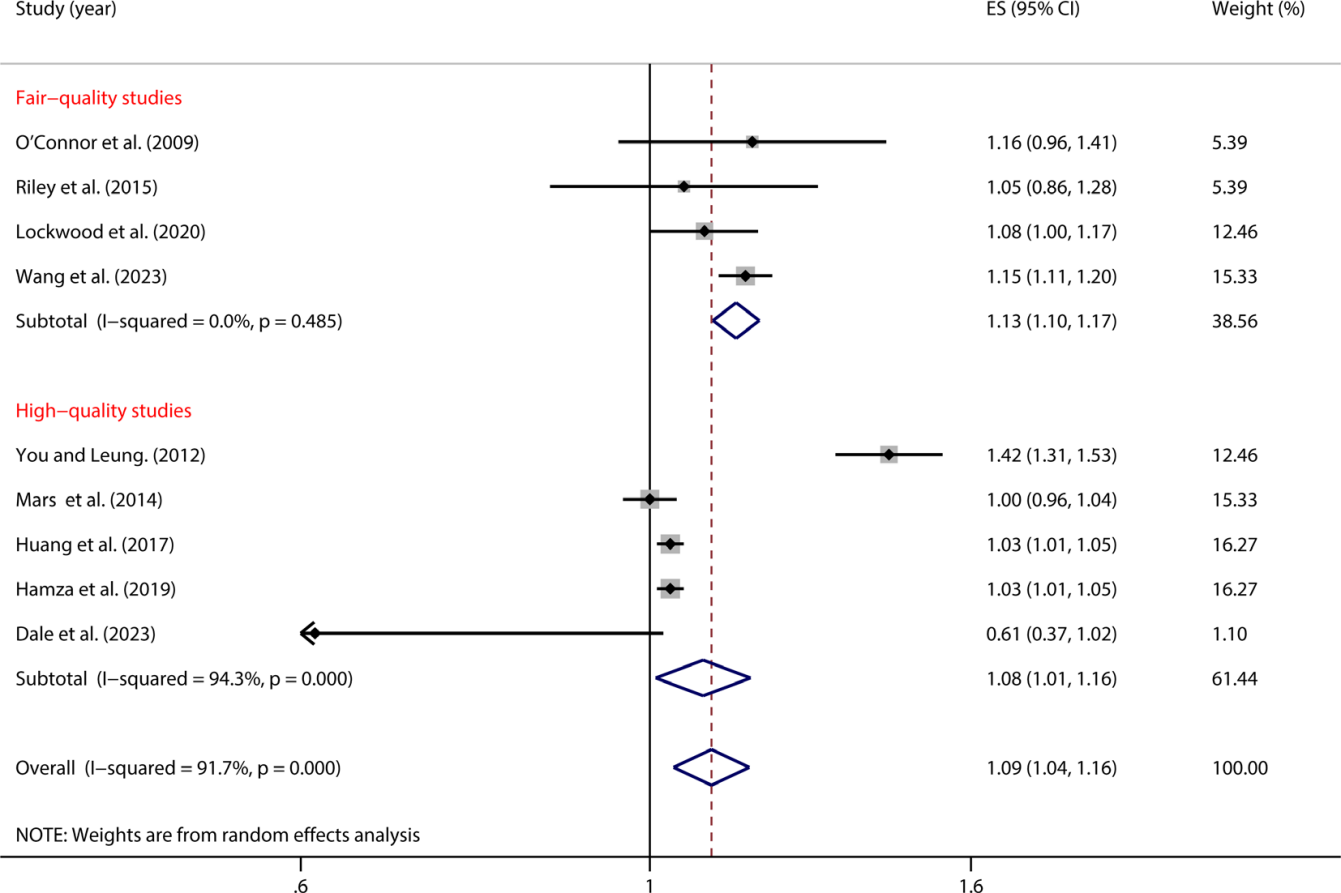
Moderation effect of quality studies on impulsivity and NSSI.

As shown in **Figure 5**, in high-quality studies, the relationship between impulsivity and NSSI was weaker (OR = 1.08, 95% *CI*: 1.01, 1.16) but still statistically significant. In contrast, in studies of Fair-quality, the relationship was stronger (OR = 1.13, 95% *CI*: 1.10, 1.17). The between-group difference was significant (*P* < 0.001), suggesting that study quality significantly moderates the relationship between impulsivity and NSSI (**Table 2**).

**Figure 6:**
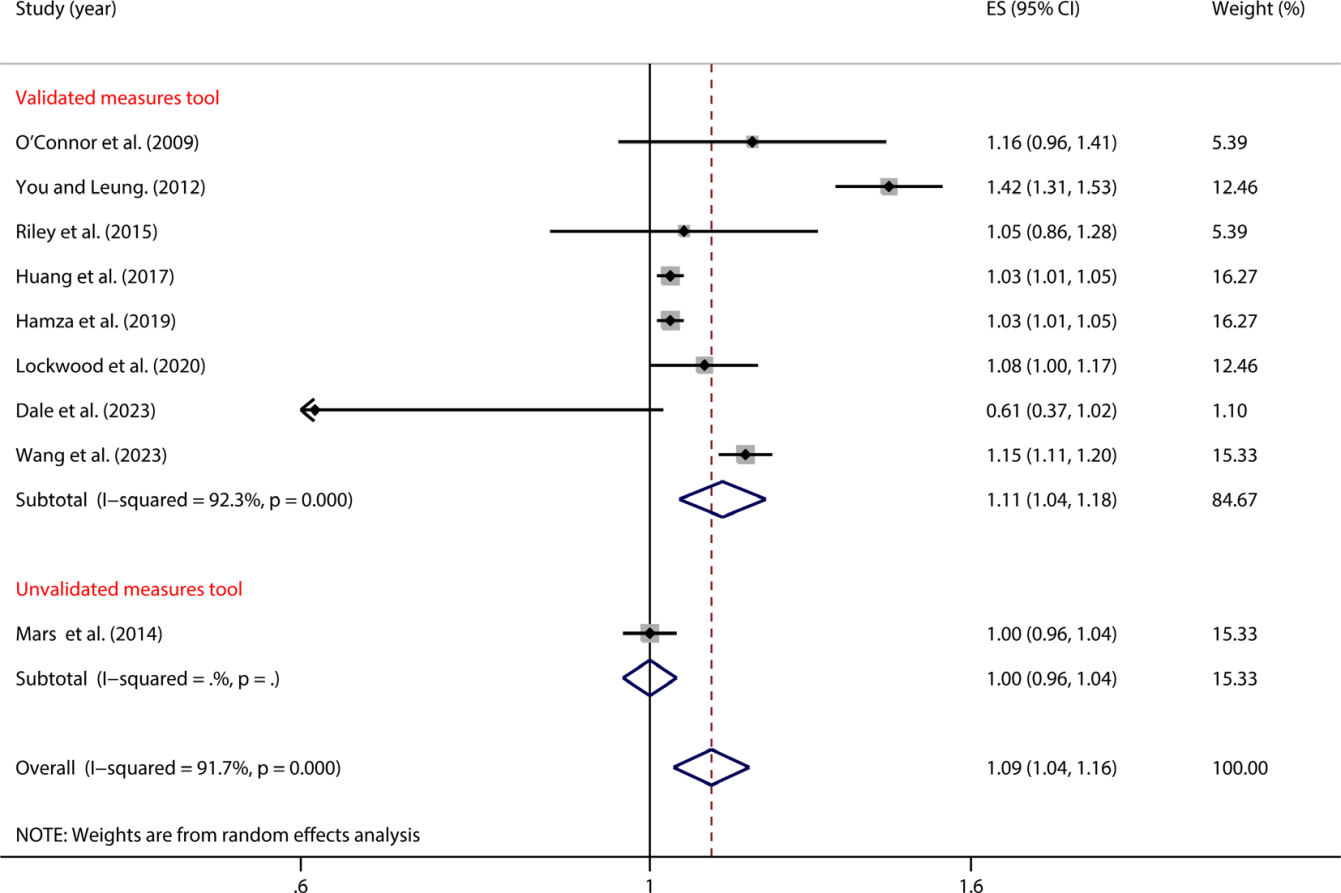
Moderation effect of Impulsivity measurement tools on impulsivity and NSSI.

As shown in **Figure 6**, in studies using validated impulsivity measurement tools, the relationship between impulsivity and NSSI was significantly associated (OR = 1.11, 95% *CI*: 1.04, 1.18). However, in studies using non-validated measurement tools, there was no significant association (OR = 1.00, 95% *CI*: 0.96, 1.04). The between-group difference was significant (*P* = 0.014), indicating that the type of impulsivity measurement tools significantly moderates the relationship between impulsivity and NSSI (**Table 2**).

**Figure 7:**
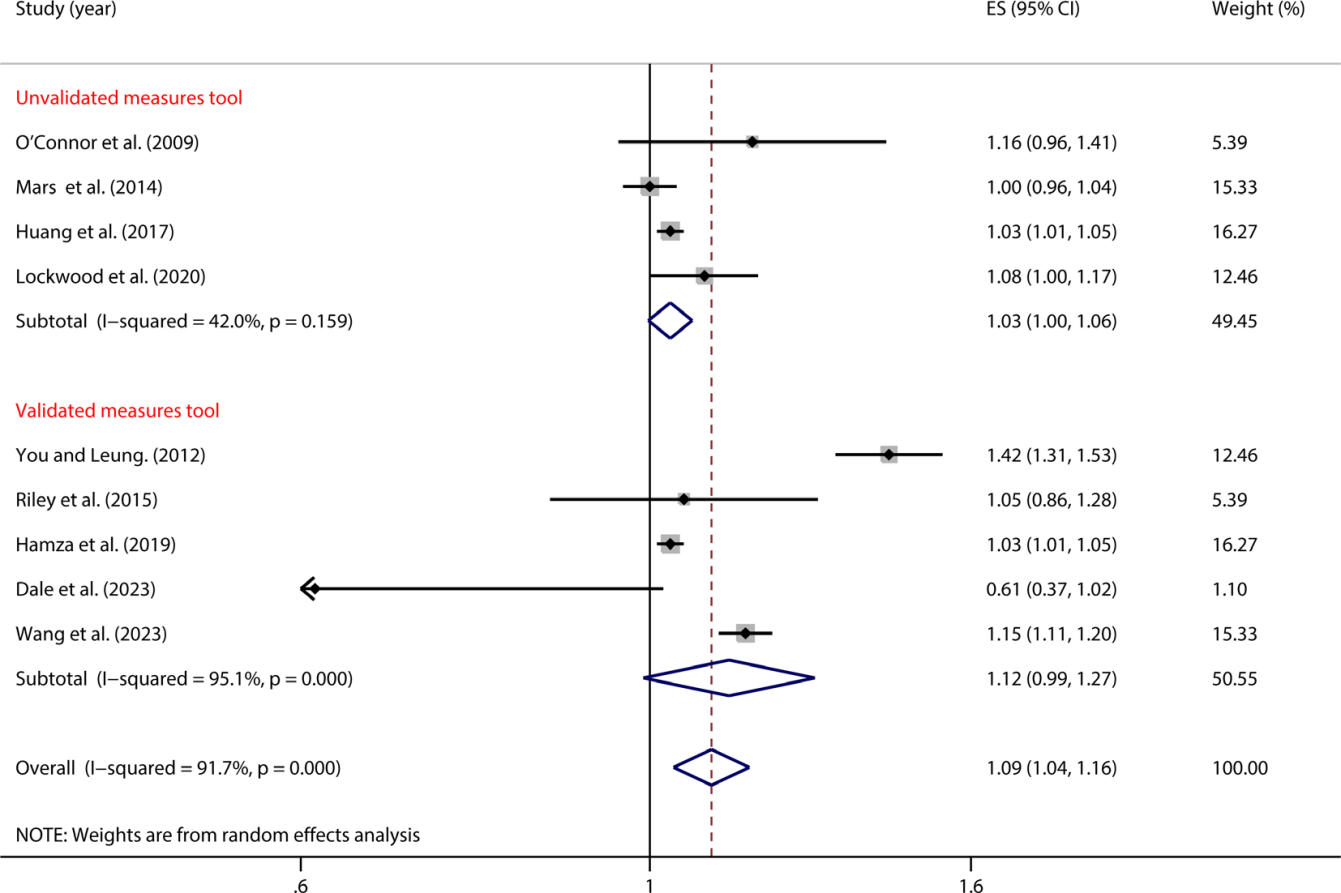
Moderation effect of NSSI measurement tools on impulsivity and NSSI.

As shown in **Figure 7**, in studies using validated NSSI measurement tools, there was no significant relationship between impulsivity and NSSI (OR = 1.12, 95% *CI*: 0.99, 1.27). Similarly, in studies using non-validated measurement tools, no significant relationship was found (OR = 1.03, 95% *CI*: 1.00, 1.06) (**Table 2**).

**Figure 8:**
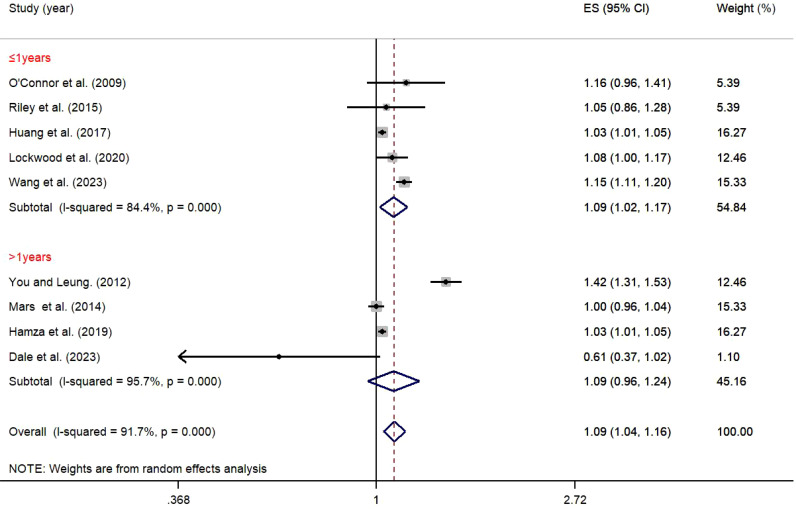
Moderation effect of follow-up period on impulsivity and NSSI.

As shown in **Figure 8**, in short-term follow-up studies (≤1 year), the relationship between impulsivity and NSSI was significant (OR = 1.09, 95% *CI*: 1.02, 1.17). However, in long-term follow-up studies (>1 year), there was no significant relationship (OR = 1.09, 95% *CI*: 0.96, 1.24). The between-group difference was not statistically significant (*P* = 0.219), suggesting that follow-up duration may not be an important factor influencing the relationship between impulsivity and NSSI (**Table 2**).

### 3.3 Sensitivity analysis

**Figure 9:**
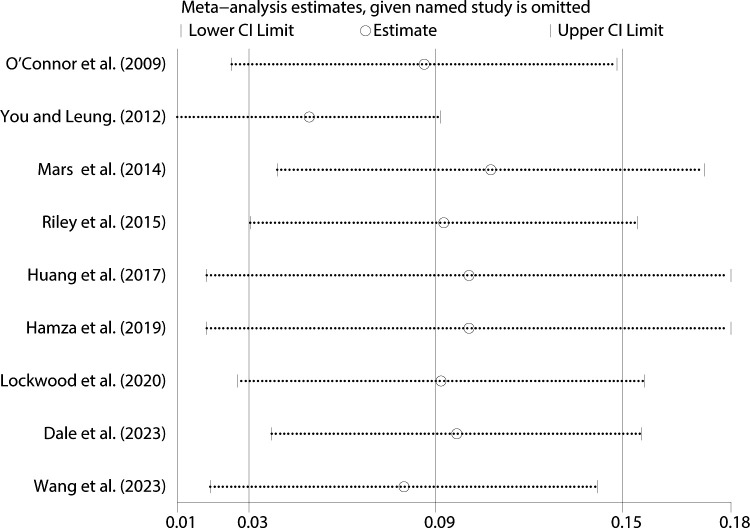
Sensitivity analysis between impulsivity and NSSI.

We conducted a sensitivity analysis to see how solid the findings were. The results of this meta-analysis are very consistent and trustworthy because the pooled effect size changed very little after individual trials were removed (**Figure 9**).

### 3.4 Publication bias

**Figure 10:**
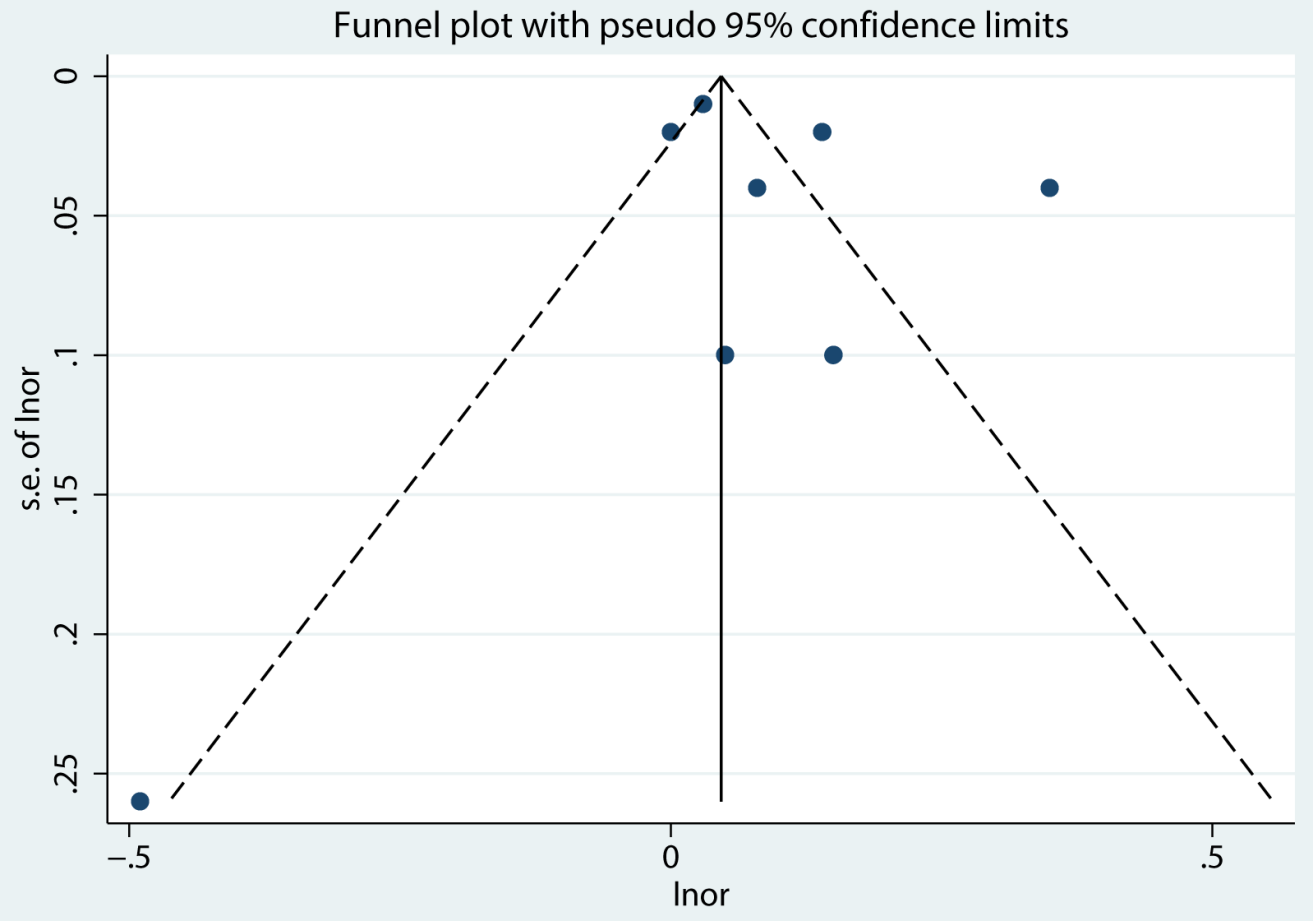
Funnel plot between impulsivity and NSSI.

**Figure 11:**
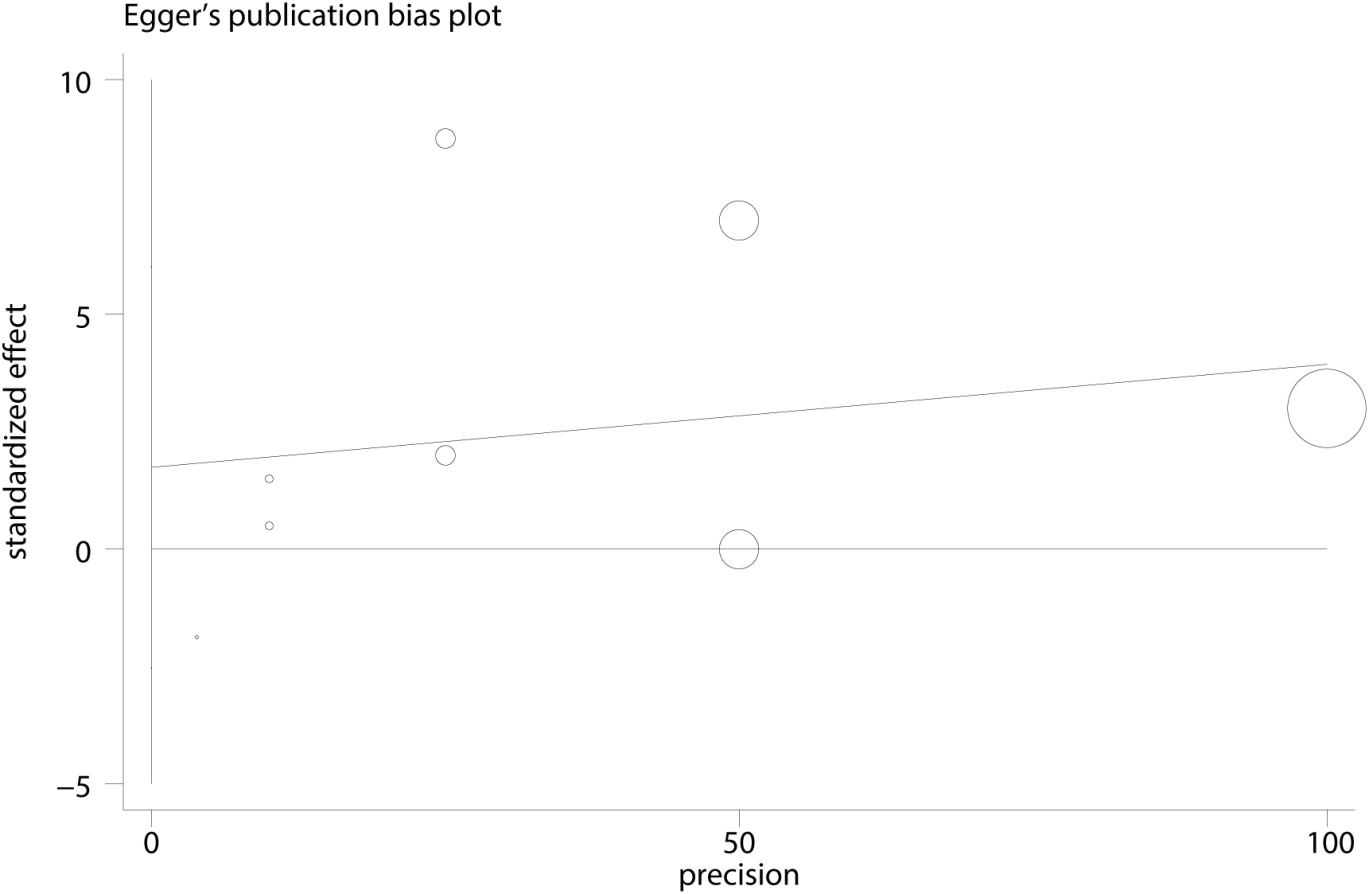
Egger plot between impulsivity and NSSI.

We built a funnel plot to evaluate the bias in the publications. There was no substantial publication bias found, and the data demonstrated that the funnel plot was generally symmetrical, indicating the reliability of the study’s findings (**Figure 10**). **Figure 11** further shows that there were no statistically significant differences (*t* = 0.66, *P* = 0.53) from the Egger’s regression test, suggesting that there is little chance of publication bias.

### 3.5 Trim and fill analysis

To further assess the robustness of the meta-analytic results and adjust for potential publication bias, we employed the Trim and Fill method. The initial analysis included 9 studies and yielded a pooled effect size of OR = 1.094 (95% CI: 1.036, 1.157, *P* < 0.001). The method imputed 2 potentially missing studies to correct for funnel plot asymmetry. After adjustment, the pooled effect size slightly decreased to OR = 1.081 (95% CI: 1.023, 1.143, *P* < 0.001). Although the adjusted estimate was marginally lower, the direction and magnitude of the effect remained highly consistent with the original analysis. Furthermore, statistical significance persisted after adjustment (*P* < 0.05), reinforcing the robustness of the association. Combined with the symmetry of the funnel plot and the results of Egger’s test as previously mentioned, these findings suggest that the overall results are stable and reliable, even after accounting for potentially unpublished studies.

## 4 Discussion

In recent years, NSSI among adolescents has become an increasingly serious public health issue worldwide (51). Meanwhile, impulsivity, as a core factor in adolescent behavioral problems, has gradually attracted considerable attention in both academic and clinical settings (31). Although a substantial body of research has explored the relationship between impulsivity and NSSI, there remains no consensus regarding the exact mechanisms underlying this association (49, 50). This study presents the first meta-analysis of longitudinal studies on impulsivity and NSSI in adolescents. We identified a significant result: adolescents with higher impulsivity exhibited a 9% greater risk of engaging in NSSI compared to those with lower impulsivity. Previous systematic reviews (33, 34) failed to identify a significant summary effect between impulsivity and NSSI, which may be attributed to the limited number of studies included in those meta-analyses (n=3 and n=7, respectively). These small sample sizes may have constrained the ability of those analyses to accurately describe the strength of the relationship between impulsivity and NSSI. In contrast, our study overcomes the limitations of previous research, which predominantly included cross-sectional designs and small sample sizes, by systematically integrating multiple longitudinal studies, thus ensuring more reliable results. Despite the methodological strengths of this study, substantial heterogeneity was observed across the included studies (*I²* = 91.7%). To explore the sources of this heterogeneity, we sub-grouped factors such as region, age, measurement tools for impulsivity and NSSI, follow-up period, and study quality. These analyses identified several significant moderators, suggesting that study-level characteristics partly explained the observed variability. In addition, residual heterogeneity may be attributed to inconsistencies in the control of confounding variables across studies, particularly with respect to baseline mental health conditions, family environment, and other contextual factors. To manage this heterogeneity, a random-effects model was applied to account for between-study variation, and sensitivity analyses confirmed the robustness of the overall effect size.

Beyond methodological differences, it is also critical to understand why impulsivity increases the likelihood of NSSI. The psychological and neurobiological mechanisms underlying this association may provide further insight into the formation of the observed pattern. The formation of this phenomenon can be explored from multiple perspectives, as outlined below. On the one hand, the Integrated Motivation-Volition (IMV) model (52) proposes the quality-stress framework, which describes the relationship between background stressors, the development of beliefs and intentions, and the translation of thoughts into actions. This model suggests that impulsivity acts as a proximal volitional moderator of self-injury, bridging the gap between intention and behavior. Impulsivity also plays a distal role in self-injury, as individuals with higher impulsivity may experience more painful and stimulating experiences over time (53). Research has shown that individuals engage in NSSI for a variety of reasons, such as emotional regulation, self-punishment, interpersonal connection, or seeking sensation (54). When influenced by negative emotions, individuals with high impulsivity may be particularly motivated to act impulsively, as the short-term benefits of emotional regulation outweigh long-term consequences (25). Given that NSSI has been shown to be an effective means of regulating negative emotions, impulsive individuals may be more prone to engage in NSSI (54). In fact, impulsive individuals may have a strong motivation to obtain the immediate benefits of NSSI, such as emotional regulation, without considering the potential long-term consequences of the behavior, such as shock, discomfort, stigma, or increased suicide risk (54). Since NSSI provides immediate relief from pain and is reinforced by negative outcomes, it increases the likelihood that individuals will engage in NSSI again in the future to regulate their negative emotional states (54). Impulsivity is associated with many forms of risky behaviors, such as alcohol and substance use, risky sexual behavior, and aggression, but impulsivity may be particularly related to engaging in NSSI (55). According to Nock’s theoretical model of the development and maintenance of NSSI, one reason individuals may choose NSSI over other forms of risky behavior is that, compared to many other behaviors that require more time to implement (e.g., obtaining drugs or alcohol), NSSI is more easily accessible (i.e., the practicality hypothesis) (56). Therefore, impulsive individuals may be particularly susceptible to engaging in NSSI because the behavior can be carried out with minimal planning or preparation.

On the other hand, adolescence, a critical developmental period, presents unique neurological characteristics. From a neurodevelopmental perspective, the development of the limbic and prefrontal systems occurs asynchronously, with the brain showing delayed prefrontal development and relatively rapid development of the limbic system. This results in reduced “top-down” higher cognitive and executive control functions, leading adolescents to exhibit higher impulsivity and poorer emotional regulation abilities (57). The prefrontal cortex, a key region in emotional processing pathways, consists of the dorsolateral, ventromedial, and orbital parts, which together process emotions. Among them, the dorsolateral prefrontal cortex (DLPFC) is most likely to be directly involved in emotional processing expression (58). Research has shown that adolescent NSSI patients exhibit reduced DLPFC activation compared to healthy controls, and the degree of DLPFC activation is negatively correlated with negative emotional responses and self-reported impulsivity. This change may drive adolescents to engage in NSSI behavior (59). Studies have found that the DLPFC is commonly targeted in clinical transcranial magnetic stimulation (TMS) to improve emotional regulation. In particular, the right dorsolateral prefrontal cortex (RDLPFC) is associated with the generation and regulation of negative emotions (60), while the left dorsolateral prefrontal cortex (LDLPFC) is linked to impulsivity (61). Abnormalities in the L/RDLPFC may mediate increased negative emotional responses and impulsivity, further promoting the occurrence of NSSI behavior.

### 4.1 The moderating role of region

The results of this study reveal a significant moderating effect of regional differences on the relationship between impulsivity and NSSI in adolescents. Specifically, adolescents in Asia who exhibit impulsivity face a significantly higher risk of engaging in NSSI compared to adolescents in non-Asian regions. Potential factors contributing to these regional differences may include several aspects. On the one hand, Asian cultures emphasize collectivism, family values, and social responsibility, which may hinder adolescents from finding appropriate ways to express and cope with impulsive behaviors (62, 63). In these cultural contexts, impulsivity may be more strongly associated with NSSI, as NSSI is often seen as a means of expressing emotional distress (64). In contrast, in non-Asian samples, particularly in Western cultures, adolescents may adopt different coping mechanisms. They may rely more on emotional regulation and seeking social support, rather than using NSSI to express emotions. Additionally, Western cultures generally have a more open approach to recognizing and addressing mental health issues, which may make it easier for adolescents to seek help and support, thus reducing the link between impulsivity and NSSI (65, 66). On the other hand, in many Asian countries, particularly in some developing nations, the availability of mental health services is limited, and adolescents may not have timely access to effective psychological interventions or treatment. This may lead adolescents with higher impulsivity to be more inclined to cope with emotional distress through NSSI. In contrast, in Western countries, especially those with well-established mental health service systems, adolescents are usually able to identify problems earlier and seek help, which may effectively reduce the relationship between impulsivity and NSSI (67, 68). Therefore, the findings of this study highlight the heightened risk of NSSI among Asian adolescents, likely due to cultural factors such as collectivism, family expectations, and limited access to mental health resources. In Asia, where mental health issues may still be stigmatized, it is critical to create safe spaces for adolescents to openly discuss their emotional struggles. This can be achieved by integrating mental health education into school systems and providing more accessible counseling services. Schools can collaborate with mental health professionals to provide culturally sensitive interventions that address both emotional well-being and the socio-cultural pressures unique to Asian adolescents.

### 4.2 The moderating role of age

Additionally, the results reveal a significant moderating effect of age differences on the relationship between impulsivity and NSSI. Specifically, the association between impulsivity and NSSI is significantly higher in middle school adolescents compared to university students. Potential factors contributing to these age differences may include the following aspects. Adolescence is a critical period for the development of self-control and emotional regulation. Particularly in middle school adolescents, due to the incomplete development of the prefrontal cortex, their ability to control impulsivity is weaker, which may make them more inclined to express internal stress and emotional conflicts through NSSI when faced with emotional distress. In contrast, university students typically possess more mature emotional regulation abilities and coping strategies. As brain development continues to mature, university students are more likely to regulate emotional issues through social interaction, seeking psychological support, or other healthy coping methods, rather than relying on NSSI as a way to release emotions (69–71). Secondly, social support and awareness of mental health may also play an important role in different age groups. University students typically have more social resources and support systems, particularly in terms of mental health, and may find it easier to seek professional help and counseling (72). Therefore, when faced with higher impulsivity, they are more likely to receive timely psychological interventions, which may reduce the association between impulsivity and NSSI. In contrast, middle school adolescents, who may lack sufficient social support, especially when dealing with emotional distress, may be more inclined to cope with stress through NSSI (73–75). Finally, age differences may also be closely related to differences in life experiences and psychological developmental stages. Adolescence is a period of emotional fluctuation, and adolescents may be more affected by peer pressure, academic stress, and other factors. These factors, combined, may make impulsivity more likely to translate into self-injurious behavior. With age and psychological maturation, university students may have developed more effective coping strategies, reducing the impact of impulsivity on NSSI (76–78). Therefore, given that middle school students are at a critical developmental stage, interventions should focus on improving emotional regulation and impulse control. Programs designed to help adolescents better manage their emotions and develop healthy coping strategies could significantly reduce the likelihood of impulsive behaviors. School-based mental health programs, which integrate emotional awareness and self-regulation skills into the daily curricula, can play a key role in promoting mental well-being and preventing self-injury among middle school students.

### 4.3 The moderating role of study quality

The results of this study highlight a significant moderating effect of research quality on the relationship between impulsivity and NSSI. Notably, the link between impulsivity and NSSI was stronger in studies rated as fair quality than in those classified as high quality. One possible explanation for this is that high-quality studies tend to employ more rigorous research designs, sample selection methods, and data analysis techniques. High quality longitudinal studies generally have stronger internal and external validity, allowing for more accurate control of potential confounding variables, such as mental health status and life events. These controls may attenuate the association between impulsivity and NSSI, leading to a smaller effect size. Studies of lower quality may have methodological limitations, such as small sample sizes, irregular data collection methods, or insufficient control of confounding variables. These factors may introduce biases in the results, making the relationship between impulsivity and NSSI appear stronger. Therefore, future research should further improve methodological standards, adopting more precise and standardized research designs to reduce potential biases and verify the true relationship between impulsivity and NSSI. Studies of fair-quality studies may have methodological limitations, such as small sample sizes, irregular data collection methods, or insufficient control of confounding variables. These factors may introduce biases in the results, making the relationship between impulsivity and NSSI appear stronger. Therefore, future research should further improve methodological standards, adopting more precise and standardized research designs to reduce potential biases and verify the true relationship between impulsivity and NSSI.

### 4.4 The moderating role of impulsivity assessment tools

Furthermore, the study found that impulsivity assessment tools significantly mediated the relationship between impulsivity and NSSI in adolescents. Specifically, adolescents assessed with validated impulsivity measures showed a higher risk of NSSI compared to those assessed with non-validated instruments. The correlation between impulsivity and NSSI could be influenced by variations in these instruments in several ways. Firstly, impulsivity assessments conducted in controlled environments may not always capture negative emotions (e.g., “When I am upset, I often act without thinking”), which is not always true in real-world settings (15, 79, 80). These findings are supported by a meta-analysis (32). Secondly, subjective perceptions of impulsive behavior (as opposed to objective impulsivity) may explain some discrepancies between experimental and self-report studies. Thus, it is possible that NSSI participants are not objectively more impulsive than non-participants, but perceive themselves as more impulsive, a bias captured by self-report measures (81). Finally, the findings from the subgroup analysis should be interpreted with caution, as it was based on only one study. This limits the ability to make definitive conclusions about the relationship between impulsivity and NSSI in this subgroup. Given these challenges, it is essential for future research to prioritize the use of standardized and psychometrically sound assessment tools. Widely adopted instruments such as the BIS-11 and the UPPS-P Impulsive Behavior Scale offer robust theoretical frameworks and strong psychometric properties. The adoption of consistent tools across studies will enhance comparability, reduce measurement-related heterogeneity, and advance a clearer understanding of the role of impulsivity in NSSI.

### 4.5 The moderating role of follow-up duration

Nonetheless, follow-up duration did not significantly moderate the relationship between impulsivity and NSSI in adolescents. Given the group’s history, this suggests that the link between impulsivity and NSSI has persisted over time. A possible explanation is that NSSI behavior is not solely driven by impulsivity but is also influenced by other factors, such as life stress, family background, and social support. Over long-term follow-up, these factors may gradually become more influential, thereby diminishing the effect of impulsivity on NSSI. Future research could explore additional potential moderating factors (e.g., individual psychological development, social support) to further elucidate the complex interactions between follow-up duration and the relationship between impulsivity and NSSI.

### 4.6 Advantages and limitations

This study is the first meta-analysis of longitudinal research examining the relationship between impulsivity and NSSI in adolescents, and it provides significant findings. Specifically, there is a significant positive correlation between impulsivity and NSSI in adolescents. Adolescents with higher impulsivity are at a 9% greater risk of engaging in NSSI compared to those with lower impulsivity. This result offers new insight into the role of impulsivity in adolescent NSSI behavior. Furthermore, the longitudinal design enables a better assessment of the causal relationship between impulsivity and NSSI, rather than merely their correlation, thereby enhancing the scientific rigor and interpretability of the findings. Consequently, the findings of this study not only expand the theoretical framework of impulsivity as a risk factor for adolescent NSSI but also provide strong support for clinical practice. In particular, timely intervention for adolescents with higher impulsivity may effectively prevent the occurrence of NSSI.

However, this systematic review and meta-analysis has some limitations. First, due to the limited number of studies available to estimate effect sizes, caution is needed when interpreting some of the subgroup analysis results. More longitudinal studies are necessary to further examine these associations. Second, we observed high heterogeneity in the main analysis of the relationship between adolescent impulsivity and NSSI, and therefore, caution should be exercised when interpreting these results. Third, much of the variability remains unexplained, as only a small number of moderating variables showed significant effects. This suggests that other potential moderating factors influencing the relationship between impulsivity and NSSI may have been overlooked in the current investigation. To gain a better understanding of the factors contributing to outcome variability and to identify the conditions most strongly associated with impulsivity and NSSI, future meta-analyses should expand their scope to include additional moderating variables. Fourth, the results may not be applicable to other demographic groups, as the study only included adolescents. The findings may not be generalizable to other populations, since children, adults, and older individuals were not included. As a result, the study’s findings may underrepresent the complexity of the relationship between impulsivity and NSSI across different age groups and fail to account for age-related differences that could influence the strength of this correlation. Future studies are encouraged to conduct age-stratified analyses or longitudinal research that includes multiple age groups to better understand how the association between impulsivity and NSSI may vary across the lifespan. Finally, it is worth noting that both impulsivity and NSSI in the included studies were primarily measured using self-report questionnaires. Although such measures are commonly used and accessible, they are subject to biases such as recall errors, social desirability, and subjective misinterpretation. These limitations may have introduced measurement-related variance into the observed associations, potentially affecting their accuracy and comparability. Future studies are encouraged to incorporate multi-method assessments, such as behavioral tasks, observer ratings, and clinician-administered interviews, in order to validate and complement self-reported data.

## 5 Conclusion

This study provides a comprehensive synthesis of the existing longitudinal literature on adolescent impulsivity and NSSI. The findings confirm that impulsivity is a robust predictor of NSSI, underscoring its value as an early identification marker for adolescents at risk. Notably, the association between impulsivity and NSSI was found to be particularly strong among middle school students and Asian adolescents, who may face unique developmental and sociocultural challenges that amplify their vulnerability. These insights carry important practical implications. First, impulsivity can serve as a valuable entry point for identifying adolescents at elevated risk of self-injury, particularly among populations that may be less likely to disclose NSSI behaviors. Second, the results highlight the urgent need to enhance adolescent mental health services by integrating targeted emotion regulation and impulse control programs within secondary school settings. These programs should be age-appropriate, culturally sensitive, and delivered in a preventive, school-based context. Furthermore, early detection systems that incorporate behavioral assessments and validated impulsivity screening tools should be established in educational and clinical environments to facilitate timely interventions. In regions such as Asia, where stigma toward mental health may limit open discussion, psychoeducation tailored to cultural norms can help destigmatize impulsivity and self-harm, promoting early help-seeking behavior. Finally, the findings call for enhanced training of educators and healthcare providers to improve recognition of early warning signs and ensure the implementation of appropriate and timely interventions. By translating these findings into practical, context-specific strategies, stakeholders can more effectively address the needs of high-risk adolescents and reduce the incidence of NSSI.

## Data Availability

The original contributions presented in the study are included in the article/**Supplementary Material**. Further inquiries can be directed to the corresponding authors.
