# Supplementary material for: Impulsivity and non-suicidal self-injury in adolescents: a systematic review and meta-analysis of longitudinal studies
Source: Front Psychiatry. 2025 May 21;16:1586922. doi: 10.3389/fpsyt.2025.1586922 (PMC12133734; doi:10.3389/fpsyt.2025.1586922)
Supplement: Supplementary file 1 [file DataSheet1.docx]

**Additional file**

**Supplementary material 1:** Search strategy used in the current systematic review and meta-analysis

**Supplementary material 2:** Methodological quality assessment of the literature

***PUBMED***

#1

"self injurious behavior"[MeSH Terms]

#2

"self-harm"[Title/Abstract] OR "self harm*"[Title/Abstract] OR "self cut*"[Title/Abstract] OR "self-destruct"[Title/Abstract] OR "nonsuicidal self injur*"[Title/Abstract] OR "non suicidal self injur*"[Title/Abstract] OR "deliberate self harm"[Title/Abstract] OR "self mutil*"[Title/Abstract] OR "overdos*"[Title/Abstract] OR "self inflicted injur*"[Title/Abstract] OR "para suicid*"[Title/Abstract] OR "parasuicid*"[Title/Abstract] OR "suicidal behav*"[Title/Abstract] OR "self harm*"[Title/Abstract] OR "self injur*"[Title/Abstract] OR "self poison*"[Title/Abstract] OR "self inflict*"[Title/Abstract]

#3

"Impulsive Behavior"[MeSH Terms]

#4

"impuls*"[Title/Abstract] OR "UPPS"[Title/Abstract] OR "Impulsive Behavior Scale"[Title/Abstract] OR "Sensation Seeking"[Title/Abstract] OR "Negative Urgency"[Title/Abstract] OR "Premeditation"[Title/Abstract] OR "Perseverance"[Title/Abstract] OR "Positive urgency"[Title/Abstract] OR "Eysenck Personality Questionnaire"[Title/Abstract] OR "Venturesomeness"[Title/Abstract] OR "Psychoticism"[Title/Abstract] OR "Barratt Impulsivity Scale"[Title/Abstract] OR "Sensation Seeking"[Title/Abstract] OR "SSS-V"[Title/Abstract] OR "ZKPQ"[Title/Abstract] OR "NEO-FFI"[Title/Abstract] OR "NEO PI-R"[Title/Abstract] OR "Neuroticism"[Title/Abstract] OR "Extraversion"[Title/Abstract] OR "Agreeableness"[Title/Abstract] OR "Conscientiousness"[Title/Abstract] OR "Tridimensional Personality Questionnaire"[Title/Abstract] OR "TPQ"[Title/Abstract] OR "Temperament and Character Inventory"[Title/Abstract] OR "TCI"[Title/Abstract] OR "Novelty seeking"[Title/Abstract] OR "Reward dependence"[Title/Abstract] OR "Harm avoidance"[Title/Abstract] OR "Cooperativeness"[Title/Abstract] OR "DAPP"[Title/Abstract] OR "MPQ"[Title/Abstract]

#5

"Longitudinal Studies"[MeSH Terms] OR "Follow-Up Studies"[MeSH Terms]

#6

"longitudinal study"[All Fields] OR "follow-up study"[All Fields] OR "prospective"[All Fields]"longitudinal"[All Fields] OR "trajector*"[All Fields] OR "course"[All Fields] OR "time point*"[All Fields]

(#1 OR #2) AND (#3 OR #4 ) AND ( #5 OR #6 )

N=424

***Web of Sci***

#1

TS=("self injurious behavior" OR "self-harm" OR "self harm*" OR "self cut*" OR "self-destruct" OR "nonsuicidal self injur*" OR "non suicidal self injur*" OR "deliberate self harm" OR "self mutil*" OR "overdos*" OR "self inflicted injur*" OR "para suicid*" OR "parasuicid*" OR "suicidal behav*" OR "self harm*" OR "self injur*" OR "self poison*" OR "self inflict*")

#2

TS= ("Impulsive Behavior" OR "impuls*" OR "UPPS" OR "Impulsive Behavior Scale" OR "Sensation Seeking" OR "Negative Urgency" OR "Premeditation" OR "Perseverance" OR "Positive urgency" OR "Eysenck Personality Questionnaire" OR "Venturesomeness" OR "Psychoticism" OR "Barratt Impulsivity Scale" OR "Sensation Seeking" OR "SSS-V" OR "ZKPQ" OR "NEO-FFI" OR "NEO PI-R" OR "Neuroticism" OR "Extraversion" OR "Agreeableness" OR "Conscientiousness" OR "Tridimensional Personality Questionnaire" OR "TPQ" OR "Temperament and Character Inventory" OR "TCI" OR "Novelty seeking" OR "Reward dependence" OR "Harm avoidance" OR "Cooperativeness" OR "DAPP" OR "MPQ")

#3

TS= ("Longitudinal Studies" OR "Follow-Up Studies" OR "longitudinal study" OR "follow-up study" OR "prospective" AND "longitudinal" OR "trajector*" OR "course" OR "time point*")

#4

#1 AND #2 AND #3

N=299

***EMBASE***

#1

"self injurious behavior"/exp

#2

"self-harm":ab,ti OR "self cut*":ab,ti OR "self-destruct":ab,ti OR "nonsuicidal self injur*":ab,ti OR "non suicidal self injur*":ab,ti OR "deliberate self harm":ab,ti OR "self mutil*":ab,ti OR "overdos*":ab,ti OR "self inflicted injur*":ab,ti OR "para suicid*":ab,ti OR "parasuicid*":ab,ti OR "suicidal behav*":ab,ti OR "self harm*":ab,ti OR "self injur*":ab,ti OR "self poison*":ab,ti OR "self inflict*":ab,ti

#3

"impulsive behavior"/exp

#4

"impuls*":ab,ti OR "UPPS":ab,ti OR "Impulsive Behavior Scale":ab,ti OR "Sensation Seeking":ab,ti OR "Negative Urgency":ab,ti OR "Premeditation":ab,ti OR "Perseverance":ab,ti OR "Positive urgency":ab,ti OR "Eysenck Personality Questionnaire":ab,ti OR "Venturesomeness":ab,ti OR "Psychoticism":ab,ti OR "Barratt Impulsivity Scale":ab,ti OR "Sensation Seeking":ab,ti OR "SSS-V":ab,ti OR "ZKPQ":ab,ti OR "NEO-FFI":ab,ti OR "NEO PI-R":ab,ti OR "Neuroticism":ab,ti OR "Extraversion":ab,ti OR "Agreeableness":ab,ti OR "Conscientiousness":ab,ti OR "Tridimensional Personality Questionnaire":ab,ti OR "TPQ":ab,ti OR "Temperament and Character Inventory":ab,ti OR "TCI":ab,ti OR "Novelty seeking":ab,ti OR "Reward dependence":ab,ti OR "Harm avoidance":ab,ti OR "Cooperativeness":ab,ti OR "DAPP":ab,ti OR "MPQ":ab,ti

#5

"longitudinal studies"/exp OR "follow-up studies"/exp

#6

"longitudinal study":ab,ti OR "follow-up study":ab,ti OR "prospective":ab,ti OR "longitudinal":ab,ti OR "trajector*":ab,ti OR "course":ab,ti OR "time point*":ab,ti

#7

(#1 OR #2) AND (#3 OR #4) AND (#5 OR #6)

N=730

**Supplementary material 2:** **Methodological quality assessment of the literature**

S2：**Newcastle-Ottawa Scales of recruited studies** (NOS)

| **Study** | **Selection** | | | | **Comparability** | **Outcome** | | | **Quality** |
| --- | --- | --- | --- | --- | --- | --- | --- | --- | --- |
|  | **Representativeness of the exposed cohort** | **Selection of the unexposed cohort** | **Ascertainment of exposure** | **Outcome of interest not present at start of study** | **Control for important factor or additional factor** | **Outcome assessment** | **Follow-up long enough for outcomes to occur** | **Adequacy of follow-up of cohorts** |  |
| O'Connor et al (2009) | ★ | ★ | — | ★ | ★★ | — | — | ★ | 6 |
| You and Leung (2012) | ★ | ★ | — | ★ | ★★ | — | ★ | ★ | 7 |
| Mars et al (2014) | ★ | ★ | — | ★ | ★★ | — | ★ | ★ | 7 |
| Riley et al (2015) | — | ★ | — | ★ | ★★ | — | ★ | ★ | 6 |
| Huang et al (2017) | ★ | ★ | — | ★ | ★★ | — | ★ | ★ | 7 |
| Hamza et al (2019) | ★ | ★ | — | ★ | ★★ | — | ★ | ★ | 7 |
| Lockwood et al (2020) | ★ | ★ | — | ★ | ★★ | — | — | ★ | 6 |
| **Study** | **Selection** | | | | **Comparability** | **Outcome** | | | **Quality** |
|  | **Representativeness of the exposed cohort** | **Selection of the unexposed cohort** | **Ascertainment of exposure** | **Outcome of interest not present at start of study** | **Control for important factor or additional factor** | **Outcome assessment** | **Follow-up long enough for outcomes to occur** | **Adequacy of follow-up of cohorts** |  |
| Dale et al (2023) | ★ | ★ | ★ | ★ | ★★ | ★ | ★ | ★ | 9 |
| Wang et al (2023) | ★ | ★ | — | ★ | ★★ | — | ★ | — | 6 |
